# Experimental and theoretical studies on induced ferromagnetism of new (1 − *x*)Na_0.5_Bi_0.5_TiO_3_ + *x*BaFeO_3−*δ*_ solid solution

**DOI:** 10.1038/s41598-021-88377-3

**Published:** 2021-04-26

**Authors:** Dang Duc Dung, Nguyen Huu Lam, Anh Duc Nguyen, Nguyen Ngoc Trung, Nguyen Van Duc, Nguyen The Hung, Yong Soo Kim, Dorj Odkhuu

**Affiliations:** 1 School of Engineering Physics, Ha Noi University of Science and Technology, 1 Dai Co Viet road, Ha Noi, Viet Nam; 2 Department of Physics, University of Ulsan, Ulsan, 680-749 Republic of Korea; 3 School of Electronics and Telecommunications, Ha Noi University of Science and Technology, 1 Dai Co Viet road, Ha Noi, Viet Nam; 4 Department of Physics, Incheon National University, Incheon, 22012 South Korea

**Keywords:** Materials science, Condensed-matter physics, Magnetic properties and materials

## Abstract

New solid solution of Na_0.5_Bi_0.5_TiO_3_ with BaFeO_3−*δ*_ materials were fabricated by sol–gel method. Analysis of X-ray diffraction patterns indicated that BaFeO_3−*δ*_ materials existed as a well solid solution and resulted in distortion the structure of host Na_0.5_Bi_0.5_TiO_3_ materials. The randomly incorporated Fe and Ba cations in the host Na_0.5_Bi_0.5_TiO_3_ crystal decreased the optical band gap from 3.11 to 2.48 eV, and induced the room-temperature ferromagnetism. Our density-functional theory calculations further suggested that both Ba for Bi/Na-site and Fe dopant, regardless of the substitutional sites, in Na_0.5_Bi_0.5_TiO_3_ lead to the induced magnetism, which is illustrated in terms of the exchange splitting between spin subbands through the crystal field theory and Jahn–Teller distortion effects. Our work proposes a simple method for fabricating lead-free ferroelectric materials with ferromagnetism property for multifunctional applications in smart electronic devices.

## Introduction

The current research trend in materials science is injecting ferromagnetism into ferroelectric materials to create next-generation smart electronic devices^1,2^. The development of ferromagnetism materials based on lead-based ferroelectric by doing transition metals, such as PbTiO_3_, has been hampered because their materials strong adverse effect on the environment and human health^3^. Ferroelectric lead-based PbTiO_3_ materials are more commonly used than lead-free ferroelectric materials, such as those based on Bi_0.5_(Na,K)_0.5_TiO_3_, (K,Na)NbO_3_, or (Ba,Ca)(Zr,Ti)O_3_^4,5^. Na_0.5_Bi_0.5_TiO_3_ as one of common lead-free ferroelectric compounds has been considered as a promising candidate that can be replacement for lead-based substances because of its stronger polarization^3^. The high polarization of Na_0.5_Bi_0.5_TiO_3_ materials is due to the lone pair effect of Bi^3+^ in comparison with that of Pb^2+^ in perovskite structures^6,7^. Therefore, injecting room-temperature ferromagnetism into lead-free ferroelectric Na_0.5_Bi_0.5_TiO_3_ is one of significant research interests.

Scholars have developed Na_0.5_Bi_0.5_TiO_3_-based materials with room-temperature ferromagnetism property by using transition metals, such as Co, Fe, Mn, or Cr^8–11^. Moreover, the solid solutions of Na_0.5_Bi_0.5_TiO_3_-based materials with BiFeO_3_ material exhibits room-temperature ferromagnetism^12^. Na_0.5_Bi_0.5_TiO_3_ materials are composed of ferrite compounds, such as CoMn_0.2_Fe_1.8_O_4_^13^. Ju et al. predicted that substituting a transition metal cation to Ti-site results in the magnetic moments because of the spin polarization of the 3*d* electrons in the transition metal^14^. However, the origin of ferromagnetism in transition metal doped lead-free ferroelectric materials were still debated. The pure Na_0.5_Bi_0.5_TiO_3_ materials exhibited the weak-ferromagnetism at room temperature which were possible explained by the surface-effect and/or self-defect^15,16^. Zhang et al*.* predicted that Na and Ti vacancies induced the magnetization rather than Bi or O vacancies^15^. Ju et al*.* reported that Na vacancies located at/near the surface of nanograins of nanocrystalline Na_0.5_Bi_0.5_TiO_3_ materials possibly displayed the ferromagnetism^16^. Such predictions were well consisted with recent obtained room temperature ferromagnetism in pure Na_0.5_Bi_0.5_TiO_3_ materials^10,11^. However, the magnetization of pure Na_0.5_Bi_0.5_TiO_3_ materials are quite small, normally less than 1 memu/g, which hinted to apply in electronics devices. Injection of transition metal into host lead-free ferroelectric Na_0.5_Bi_0.5_TiO_3_ materials possibly enhanced the magnetization up to ~ 9 memu/g^10^. Unlikely the picture of ferromagnetism at room temperature of pure Na_0.5_Bi_0.5_TiO_3_, a various magnetism sources were injected to lead-free ferroelectric materials which resulted in the room temperature ferromagnetism; such as the O-vacancies (in case of Cr-doped Na_0.5_Bi_0.5_TiO_3_), magnetic clusters (in case of Co-doped Na_0.5_Bi_0.5_TiO_3_), or interaction of magnetic cations through oxygen vacancies as intrinsic phenomenon (in case of Fe-, Mn-doped Na_0.5_Bi_0.5_TiO_3_)^8–11^. Therefore, the origin of room temperature ferromagnetism needs to be deep understood to control the magnetization for smart-electronic devices application.

Thank to well solid solution of Na_0.5_Bi_0.5_TiO_3_ material with various type of *AB*O_3_ dopant materials, the physical properties of host Na_0.5_Bi_0.5_TiO_3_ materials were enhanced^17–27^. Rahman et al. reported that both ferroelectric and piezoelectric properties of Na_0.5_Bi_0.5_TiO_3_ increased via solid solution of BaZrO_3_ where the both remanent polarization and piezoelectric constant increased from 22 μC/cm^2^ and 60 pC/N for pure Na_0.5_Bi_0.5_TiO_3_ to 30 μC/cm^2^ and 112 pC/N for 4 mol% BaZrO_3_ solid solution in Na_0.5_Bi_0.5_TiO_3_ materials^17^. Yang et al. reported that the (Ba_0.7_Ca_0.3_)TiO_3_ solid solution in host Na_0.5_Bi_0.5_TiO_3_ materials resulted in greatly lowered coercive field without degrading remanent polarization^18^. Bai et al. reported that the Bi(*Me*_0.5_Ti_0.5_)O_3_ (*Me* = Zn, Ni, Mg, Co)-modified Na_0.5_Bi_0.5_TiO_3_ materials displayed the large strain response (> 0.3%) with a high normalized strain *S*_max_/*E*_max_ (> 550 pm/V)^19^. Zhou et al*.* reported that BaNb_2_O_6_ diffused into lattice of Na_0.5_Bi_0.5_TiO_3_ to form a solid solution resulted in enhancement of the dielectric properties of host Na_0.5_Bi_0.5_TiO_3_ materials^20^. Kaswan et al. reported on ferromagnetism in Bi_0.5_Na_0.5_TiO_3_-Bi_0.8_Ba_0.2_FeO_3_ composite materials^21^. Pattanayak et al. observed the ferromagnetic properties of a BaFe_12_O_19_-modified Bi_0.5_Na_0.5_TiO_3_ system^22^. Recently, Singh et al. reported on the ferromagnetic properties of Bi_0.5_Na_0.5_TiO_3_ materials induced by the addition of LaFeO_3_ as solid solution^23^. In addition, the magnetic properties of Na_0.5_Bi_0.5_TiO_3_ materials were found to be strong enhancement such magnetization via solid solution with various impurities materials such as ilmenite-type materials (e.g. MnTiO_3_, NiTiO_3_, FeTiO_3_, or CoTiO_3_), or perovskite-type materials (e.g. MgFeO_3−δ_, SrFeO_3−δ_, CaFeO_3−δ_, SrMnO_3−δ_, CaMnO_3−δ_, BaMnO_3−δ_, MgMnO_3−δ_, SrCoO_3−δ_, MgCoO_3−δ_, BaCoO_3−δ_, or CaCoO_3−δ_)^24–38^. The double perovskite-type structural materials containing the transition metals (e.g. Bi(Ti_0.5_Fe_0.5_)O_3−δ_, Bi(Ti_0.5_Mn_0.5_)O_3−δ_, Bi(Ti_0.5_Co_0.5_)O_3−δ_, or Bi(Ti_0.5_Ni_0.5_)O_3−δ_) were also reported to enhance the magnetic properties of Na_0.5_Bi_0.5_TiO_3_ materials when their materials were solid solution into host materials^39–42^. The magnetization of modified- Na_0.5_Bi_0.5_TiO_3_ samples via impurities of ilmenite-type materials, perovskite-type or double perovskite-type structural materials were found to have large magnetization moment which were compared with single transition metal dopants^8–11,24–42^.

Among alkaline-earth iron perovskite *Ae*FeO_3−δ_ family (*Ae* = Ba, Ca, Sr, and Mg), BaFeO_3−δ_ is one of interesting materials because its ferromagnetic domains could be controllable by an applied magnetic field^43^. BaFeO_3−δ_ materials exhibited complex of phase such as monoclinic, rhombohedral, pseudo-cubic and cubic which depended on the valence state of Fe and transition between them^44^. Mori et al. reported that BaFeO_3−δ_ compounds existed in many forms such hexagonal phase in a wide range of oxygen content BaFeO_2.63–2.92_ while other phase has exhibited such triclinic I, BaFeO_2.50_; triclinic II, BaFeO_2.64–2.67_; rhombohedral I and II, BaFeO_2.62–2.64_; and tetragonal, BaFeO_2.75–2.81_^45^. The cubic perovskite BaFeO_3_ with Fe^4+^ state has *A*-type spiral spin structure as ferromagnetism below 111 K^46^. Clemens et al. reported that BaFeO_2.5_ materials with Fe^3+^ state exhibited the *G*-type antiferromagnetic structure with Neel temperature of 720 K^47^. Delattre et al. reported that the BaFeO_2.8_ with orthorhombic structural exhibited the strong couple antiferromagnet^48^. Theoretical simulation predicted that the BaFeO_2_ materials has tetragonal symmetry and the *G*-type antiferromagnetic spin configuration^49^. The highest ferromagnetic ordering around 235 K in BaFeO_3−δ_ were obtained for thin film growing on the SrTiO_3_ substrate^50^. Recently, the new system of (1-*x*)Na_0.5_Bi_0.5_TiO_3_ + *xAe*FeO_3−δ_ (*Ae* = Sr, Ca, and Mg) materials as solid solution were successful fabricated by using the sol–gel technique^28–30^. The results provided that the impurities cation (such as Sr, Mg) and Fe random incorporated with (Bi,Na)-site and Ti-site, respectively, were exhibited the strong ferromagnetism at room temperature where the magnetization were found to great enhancement than that of single transition metal dopants which were possible resulted from co-modification at *A*-site via alkaline-earth and Fe cation at *B*-site of host Na_0.5_Bi_0.5_TiO_3_^28–30^. In the periodic table of elements, Ba is the largest radius in alkaline earth metals, thus, we expected that the co-modification of Ba cations at *A*-site and Fe cations at *B*-site, respectively, of host Na_0.5_Bi_0.5_TiO_3_ materials were resulted exhibition large magnetization during solid solution of BaFeO_3−δ_ into Na_0.5_Bi_0.5_TiO_3_ materials.

In this work, new system (1-*x*)Na_0.5_Bi_0.5_TiO_3_ + *x*BaFeO_3−*δ*_ materials as solid solution were fabricated by sol–gel method. The BaFeO_3−*δ*_ materials were well solid solution into the host Na_0.5_Bi_0.5_TiO_3_ materials through diffusion and random incorporation of Ba and Fe cations with host lattice of Na_0.5_Bi_0.5_TiO_3_ materials. The structural distortion and reduced optical band gap of host Na_0.5_Bi_0.5_TiO_3_ materials were obtained. The complex magnetic properties of BaFeO_3−*δ*_-modified Na_0.5_Bi_0.5_TiO_3_ materials was obtained as function of BaFeO_3−*δ*_ amounts addition.

## Results and discussion

**Figure 1:**
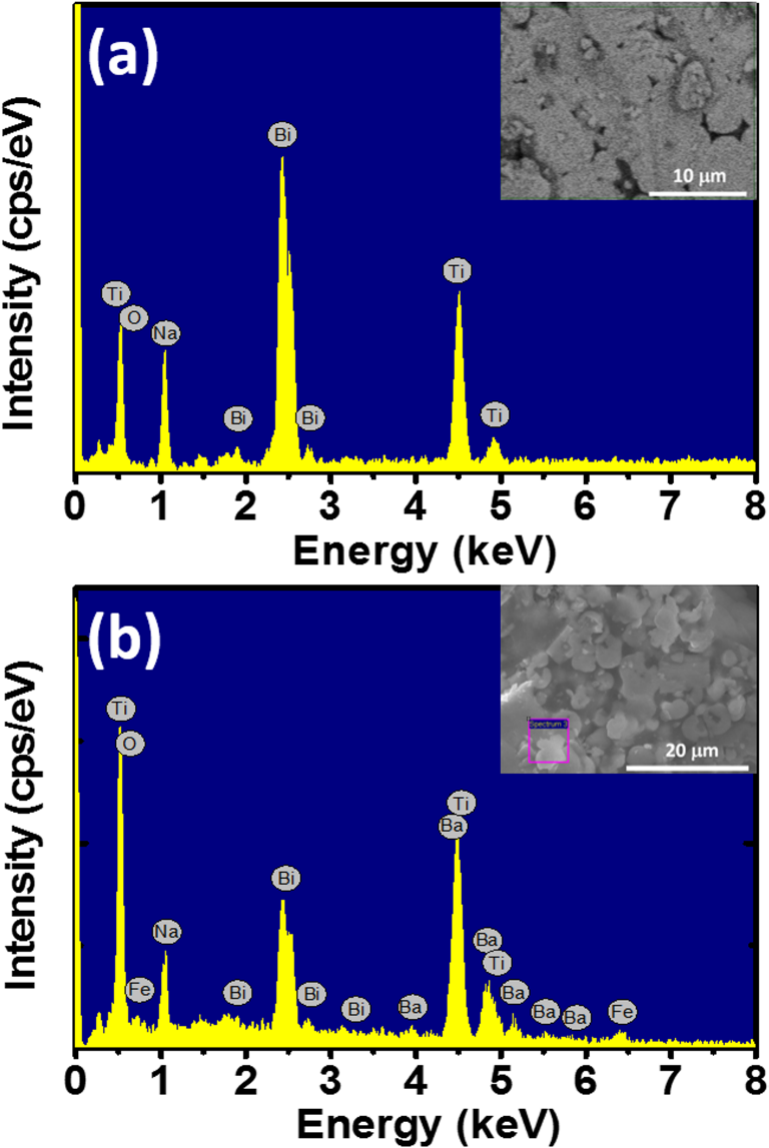
The EDS spectral of **(a)** pure Na_0.5_Bi_0.5_TiO_3_ materials, and **(b)** BaFeO_3−*δ*_-modified Na_0.5_Bi_0.5_TiO_3_ materials with 5 mol% BaFeO_3−δ_ as solid solution. The inset of each figure shown the selected area for composition characterization.

Figure 1a,b shows the EDS spectral of pure Na_0.5_Bi_0.5_TiO_3_ samples and BaFeO_3−δ_-modified Na_0.5_Bi_0.5_TiO_3_ sample with 5 mol.% BaFeO_3−δ_, respectively. The inset of each figure showed the selected area for EDS elements characterization. All expectational elements such Bi, Na, Ti and O were obtained in EDS spectral of pure Na_0.5_Bi_0.5_TiO_3_ samples, as shown in Fig. 1a. The addition of the Ba and Fe peaks were showed in the EDS spectral of BaFeO_3−δ_-modified Bi_0.5_Na_0.5_TiO_3_ samples, as expected, which were presented at the Fig. 1b. The results provided that the BaFeO_3−δ_ impurities existed in our samples.

**Figure 2:**
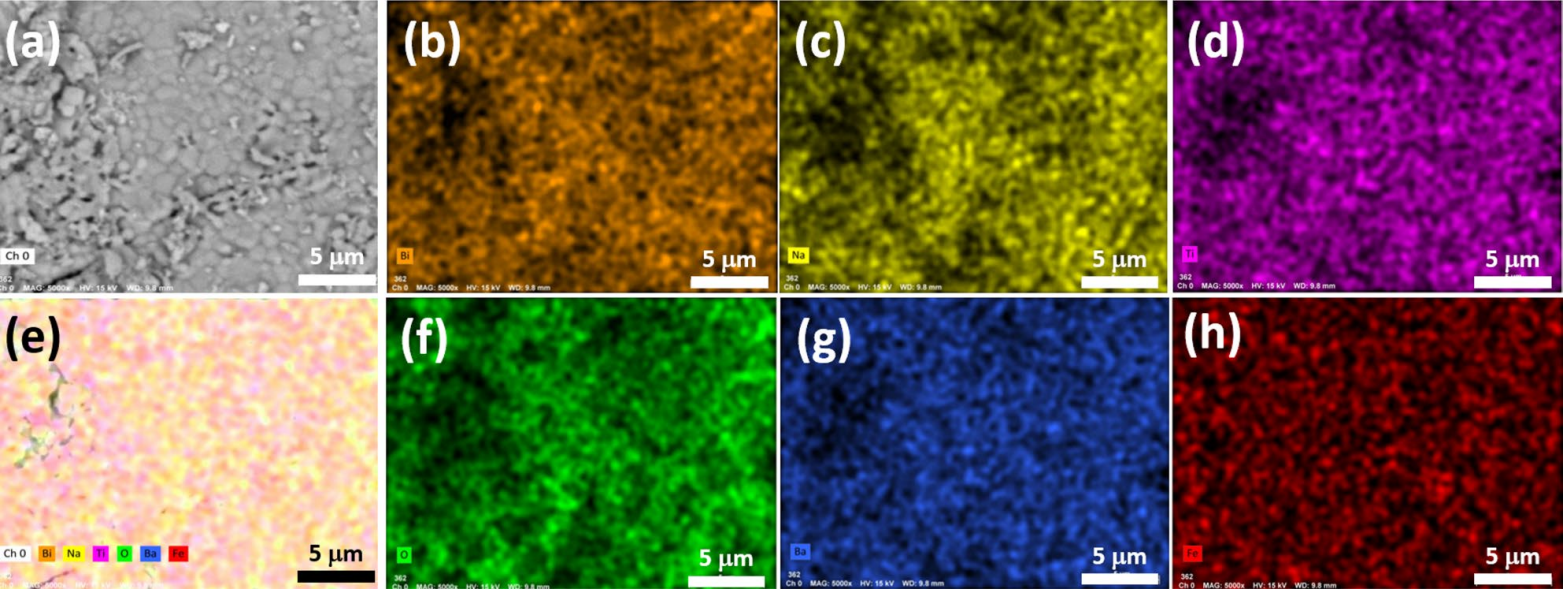
**(a)** Selected area for the chemical mapping of 9 mol.% BaFeO_3−δ_-modified Na_0.5_Bi_0.5_TiO_3_ materials; **(b)** total chemical element distribution in samples; and (c)–(h) partial chemical element maps of Bi, Na, Ti, O, Ba, and Fe.

The chemical maps of Bi_0.5_Na_0.5_TiO_3_ materials modified with 9 mol.% BaFeO_3−δ_ were analyzed. The distribution of impurity elements in the Na_0.5_Bi_0.5_TiO_3_ materials modified with 9 mol.% BaFeO_3−δ_ is shown in Fig. 2. The surface morphology of the area selected for chemical mapping is shown in Fig. 2a, whereas Fig. 2b presents the total contribution of all of the chemical elements in the sample. The partial chemical maps of Bi, Na, Ti, O, Ba, and Fe elements are shown in Fig. 2c–h, respectively. The results clearly demonstrated that the constituent chemical elements were homogenously dispersed in the sample.

**Figure 3:**
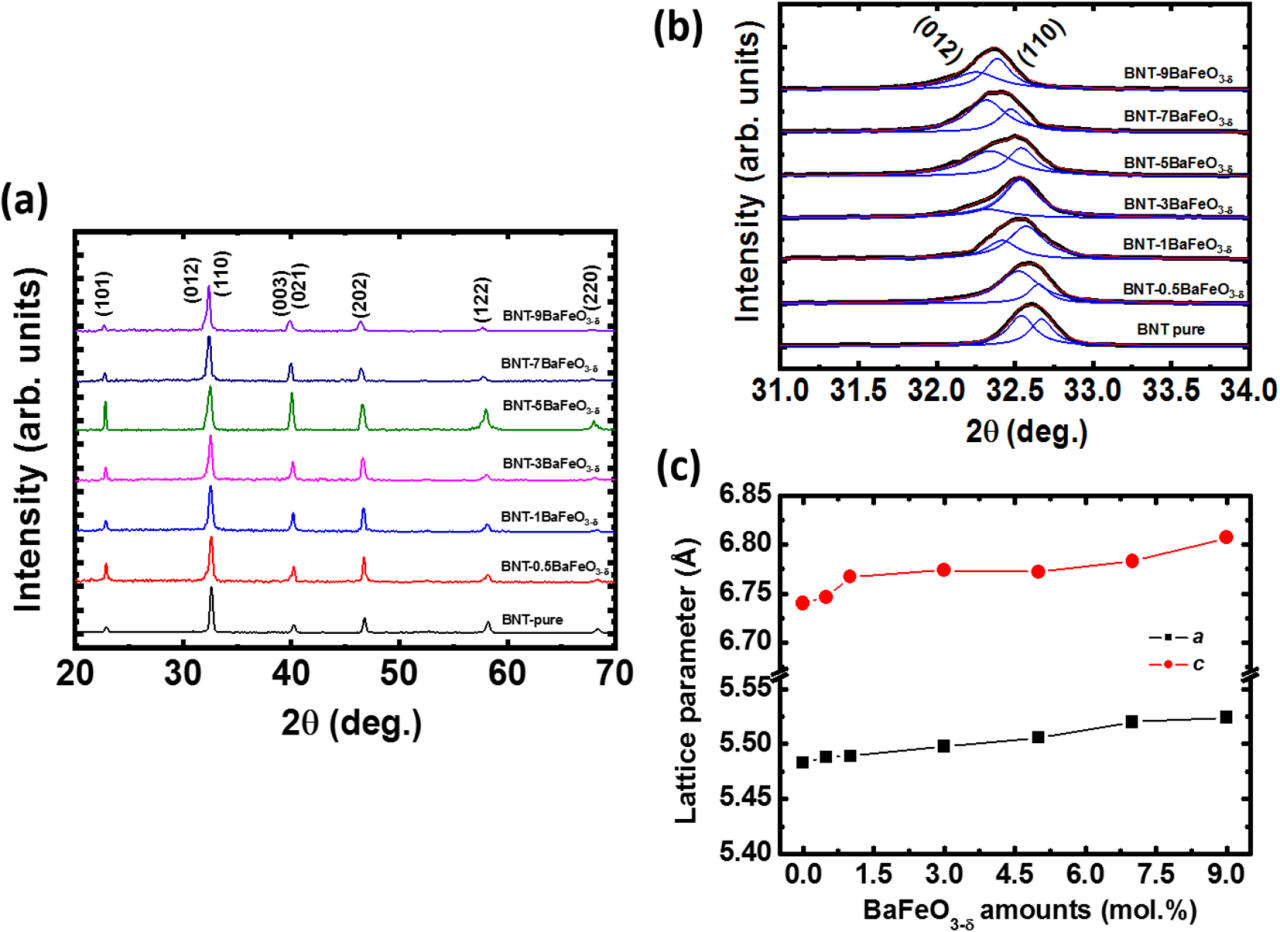
**(a)** X-ray diffraction pattern of BaFeO_3−*δ*_ solid solution into Na_0.5_Bi_0.5_TiO_3_ with various concentrations within the 2θ range of 20° to 70°; **(b)** magnified X-ray diffraction within 2θ range of 31°–34° for comparing setline (012)/(110) peaks; and **(c)** dependence of the lattice parameters of pure Bi_0.5_Na_0.5_TiO_3_ and BaFeO_3−*δ*_*-*modified Bi_0.5_Na_0.5_TiO_3_ samples on the amounts of BaFeO_3−δ_ solid solution.

Figure 3 (a) shows the X-ray diffraction patterns of pure Na_0.5_Bi_0.5_TiO_3_ and BaFeO_3−δ_-modified Na_0.5_Bi_0.5_TiO_3_ with various BaFeO_3−δ_ concentrations. On the basis of diffraction peak position and relative to intensity, all samples were indexed to a perovskite structure with the rhombohedral symmetry of the Na_0.5_Bi_0.5_TiO_3_ compound (JCPDS card no. 00–036-0340, space group R3c). In addition, the impurities phase or phase segregation was not founded in the X-ray diffraction patterns. All X-ray diffraction pattern of BaFeO_3−δ_-modified Na_0.5_Bi_0.5_TiO_3_ samples were indexed to follow the structural of Na_0.5_Bi_0.5_TiO_3_ compound. The results indicated that BaFeO_3−δ_ materials exhibited a well solid solution in Na_0.5_Bi_0.5_TiO_3_ materials. In other word, the Ba and Fe cations were diffused to randomly incorporate with host lattice of Na_0.5_Bi_0.5_TiO_3_ compound as solid solution. In order to character the influence of Ba and Fe into host crystalline of Na_0.5_Bi_0.5_TiO_3_ compound, the diffraction angle of pure Na_0.5_Bi_0.5_TiO_3_ and BaFeO_3−δ_-modified Bi_0.5_Na_0.5_TiO_3_ samples was magnified within 31.0° to 34.0° for setline (012)/(110) peaks, as shown in Fig. 3b. The setline peaks were overloaded together which were distinguished via Lorentz fitting, as shown in dot line of Fig. 3b. The results clearly indicated that the diffraction peaks of Na_0.5_Bi_0.5_TiO_3_ materials trended to shift to lower diffraction angle as increasing the BaFeO_3−δ_ amounts, which provided the evident for expansion of lattice parameter. Furthermore, the lattice parameters *a* and *c* of the pure Bi_0.5_Na_0.5_TiO_3_ and the BaFeO_3–δ_-modified Bi_0.5_Na_0.5_TiO_3_ as a function of BaFeO_3–δ_ addition amounts are shown in Fig. 3c. The results show that the distorted lattice parameters of the Bi_0.5_Na_0.5_TiO_3_ compound are not a linear function of the concentrations of the BaFeO_3–δ_ solid solution, which showed complex lattice parameter distortion. This result could be attributed to the different radii of Ba and Fe cations in the additives and that of Bi, Na, and Ti incorporated randomly in the lattice of the host Na_0.5_Bi_0.5_TiO_3_ materials. Based on the Shamon’ reported, the radius of Ba^2+^ and Fe^2+/3+^ cations were 1.61 Å and 0.645 Å/0.780 Å, respectively, while the radius of Bi^3+^, Na^+^ and Ti^4+^ cations were 1.17 Å, 1.39 Å and 0.605 Å, respectively^51^. Therefore, the Fe cations diffused to substitute for Ti-sites in perovskite structural of Na_0.5_Bi_0.5_TiO_3_ crystal, resulted in expansion of the lattice. The fact that the radius of Ba^2+^ cations are larger than that of both Bi^3+^ and Na^+^ cations were also reflected by expanding the lattice parameter of host Na_0.5_Bi_0.5_TiO_3_ compound. However, we noted that the oxygen vacancies were generated due to unbalance of valence states of Fe^2+/3+^ and Ti^4+^ at *B*-site and Ba^2+^ for Bi^3+^ at *A*-site. In addition, the Na vacancies were created when Ba^2+^ substitute Na^+^. The oxygen vacancies (¤) has radius of 1.31 Å which were smaller than that of oxygen anion (O^2-^) of 1.4 Å^52^. Therefore, the existence of oxygen vacancies in the structure led to reduction of the lattice parameter. The structural distortion of Na_0.5_Bi_0.5_TiO_3_ materials was due to co-modification at *A*- and *B*-site via alkali earth and transition metal, respectively, which was consistent with recently reported^28–30^. In other word, the X-ray diffraction characterization of BaFeO_3−δ_-modified Na_0.5_Bi_0.5_TiO_3_ samples provided that the BaFeO_3−δ_ materials were well solid solution into host Na_0.5_Bi_0.5_TiO_3_ materials.

**Figure 4:**
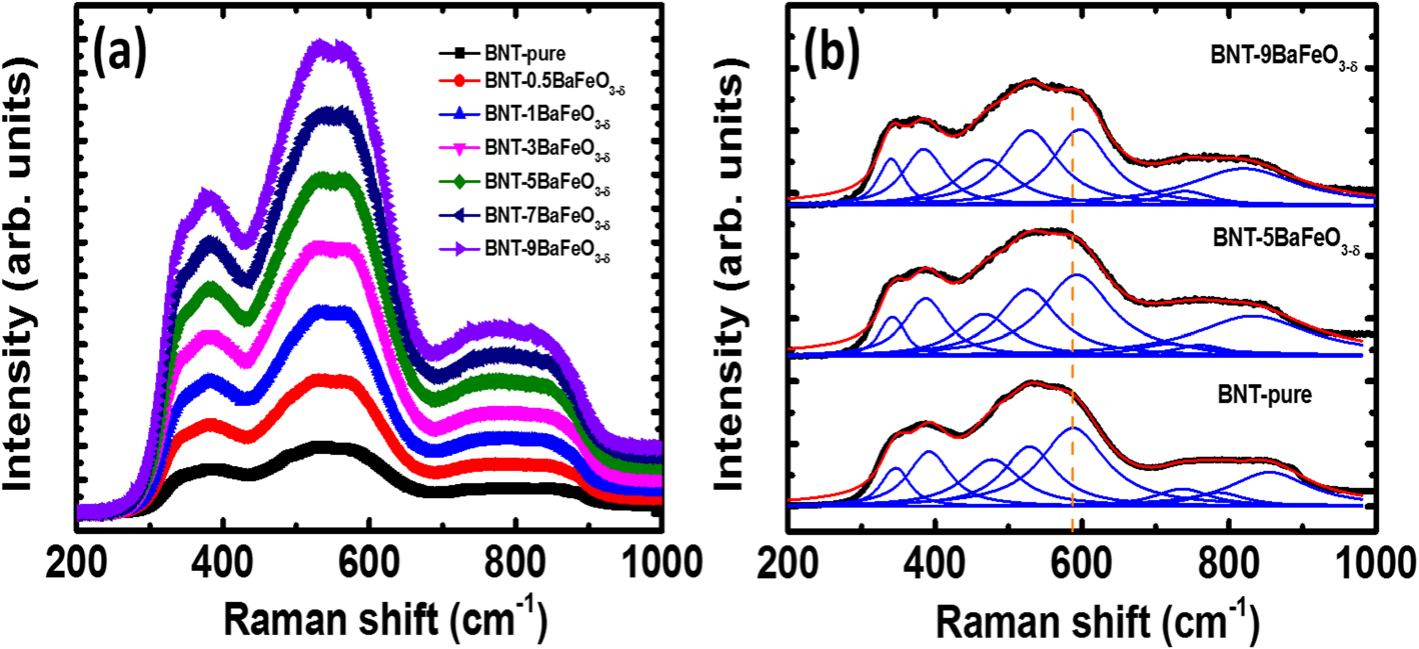
**(a)** Raman scattering spectra of BaFeO_3−*δ*_ solid solution into Na_0.5_Bi_0.5_TiO_3_ with various concentrations from 200 cm^−1^ to 1000 cm^−1^; and **(b)** deconvolution Raman peaks of pure Na_0.5_Bi_0.5_TiO_3_ and 5 and 9 mol% BaFeO_3−*δ*_ solid solution into Na_0.5_Bi_0.5_TiO_3_.

Figure 4a show the Raman scattering of pure Na_0.5_Bi_0.5_TiO_3_ materials and BaFeO_3−δ_-modified Na_0.5_Bi_0.5_TiO_3_ materials with various of BaFeO_3−−δ_ concentration as solid solutions at room temperature. The results provide that the shape of Raman scattering spectra seem to be unchanged in comparison between that of pure Na_0.5_Bi_0.5_TiO_3_ materials and that of BaFeO_3−δ_-modified Na_0.5_Bi_0.5_TiO_3_ s materials. In the wave number ranging from 300 cm^−1^ to 1000 cm^−1^, the Raman spectra were possible divided into three main regions and they overlapped each other. The three main band regions were in the range of 300–450 cm^−1^, 450–700 cm^−1^ and 700–1000 cm^−1^, respectively. The combination of experimental investigation and first principles density functional theoretical calculation for Raman vibration modes of Na_0.5_Bi_0.5_TiO_3_ materials exhibited that the lowest frequency modes in range of 246–401 cm^−1^ are dominated by TiO_6_ vibrations, and the higher frequency modes in the range 413–826 cm^−1^ are primarily associated with the oxygen atoms vibrations^53^. The random occupation at *A*-sites of Bi and Na resulted in overlap of Raman scattering peaks in range of 109–134 cm^−1^ and 155–187 cm^−1^, which were originated from Bi-O and Na–O vibration modes, respectively^53^. In addition, Chen et al. reported that the vibration of Ti–O bonds is related to the wave number in range of 200–400 cm^−1^ while the vibration of TiO_6_ octahedra is assigned to the wave number regions from 450 cm^−1^ to 700 cm^−1^^54^. The overlapping of Raman scattering modes was hard to characterize the influence of Ba and Fe into vibration modes of host lattice Na_0.5_Bi_0.5_TiO_3_ materials. Therefore, we tried to distinguish the Raman scattering modes via the fitting with Lorentz functions (with correction of fitting over 0.99). The deconvolution Raman scattering modes (blue line) of pure Na_0.5_Bi_0.5_TiO_3_ samples and BaFeO_3−δ_-modified Na_0.5_Bi_0.5_TiO_3_ samples with example for 5 and 9 mol% BaFeO_3−δ_, as shown in Fig. 4b. The eight Raman scattering vibrational modes were obtained for both pure Na_0.5_Bi_0.5_TiO_3_ and BaFeO_3−δ_-modified Na_0.5_Bi_0.5_TiO_3_ samples. The results were well consistent with recently observation in vibration of Raman scattering modes of perovskite-type structural *Ae*FeO_3−δ_ family-modified Na_0.5_Bi_0.5_TiO_3_ materials^28–30^. The vibration modes at around 595 cm^−1^ (red dot line marked in the Fig. 4b) trended to shift to high frequency, which were suggested to be related to distorted structure of (Ti,Fe)O_6_ framework and/or effective mass effect because of difference between the radius and mass of impurities Fe and host Ti at *B*-site^28–30^. In other word, the shifted Raman scattering modes confirmed the substitution of Ba and Fe into the host lattice of Na_0.5_Bi_0.5_TiO_3_ materials.

**Figure 5:**
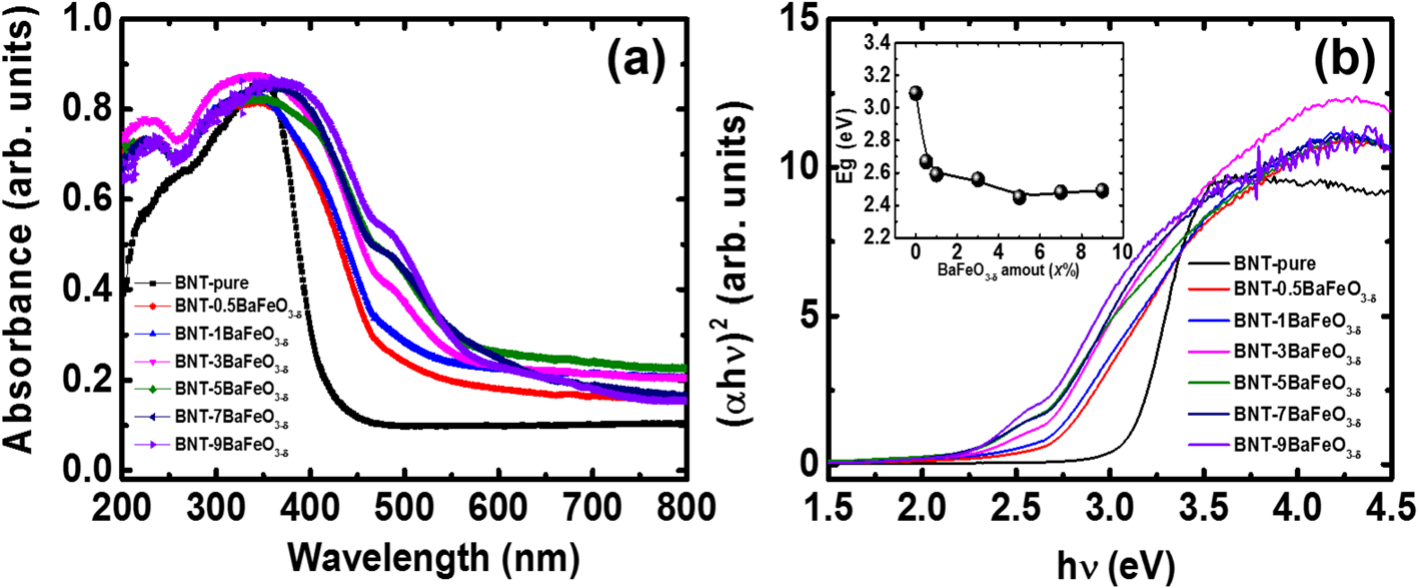
**(a)** UV–Vis absorption spectra of BaFeO_3−*δ*_-modified Na_0.5_Bi_0.5_TiO_3_ samples as a function of BaFeO_3−*δ*_ concentration; and **(b)** the (α*hν*)^2^ proposal with photon energy (*hν*) of Na_0.5_Bi_0.5_TiO_3_ samples as a function of the amount of BaFeO_3−*δ*_ added. The inset of **(b)** shows the optical band gap *E*_g_ value of Na_0.5_Bi_0.5_TiO_3_ samples as a function of the amount of BaFeO_3−*δ*_ added.

Figure 5a shows the optical absorption spectra of pure Na_0.5_Bi_0.5_TiO_3_ and BaFeO_3−δ_-modified Na_0.5_Bi_0.5_TiO_3_ with various BaFeO_3−δ_ concentrations. Pure Na_0.5_Bi_0.5_TiO_3_ samples exhibited a single absorbance edge, consistent with the reported optical properties of Na_0.5_Bi_0.5_TiO_3_ materials^9–11,55^. However, pure Na_0.5_Bi_0.5_TiO_3_ materials exhibited the unsharp transition which were tailored with slightly tail. The small tail at long wavelength in absorbance spectroscopy of pure Na_0.5_Bi_0.5_TiO_3_ materials were suggested to be related with self-defect and/or surface effect cause of unsaturation bonding pair of atoms at the surface^55,56^. The addition BaFeO_3−δ_ to Na_0.5_Bi_0.5_TiO_3_ material as solid solution led to a red shift of the absorbance edge. The appearance of peaks around 485 nm in the absorbance spectra of BaFeO_3−δ_-modified Na_0.5_Bi_0.5_TiO_3_ materials indicated the new local states of Fe cations in the middle electronic band structure of Na_0.5_Bi_0.5_TiO_3_ materials^28–30,57^. Optical band gap (*E*_g_) values of pure Na_0.5_Bi_0.5_TiO_3_ and BaFeO_3−δ_-modified Na_0.5_Bi_0.5_TiO_3_ materials were calculated using the plot of (*αhν*)^2^ versus photon energy *hν,* as shown in Fig. 5b, where *α, h* and *ν* are the absorbance coefficient, the Planck constant and the frequency, respectively. The band gap energy of pure Na_0.5_Bi_0.5_TiO_3_ materials were estimated to be approximately 3.09 eV, whereas that of BaFeO_3−δ_-modified Na_0.5_Bi_0.5_TiO_3_ materials exhibited value of 2.48 eV for 9 mol. % BaFeO_3−δ_ solid solution in host Na_0.5_Bi_0.5_TiO_3_ materials. The detail dependence of *E*_g_ values of BaFeO_3−δ_-modified Na_0.5_Bi_0.5_TiO_3_ compound as function of BaFeO_3−δ_ concentration is shown in the inset of Fig. 5b. The optical band gap of Na_0.5_Bi_0.5_TiO_3_ material in which the Ti-sites substituted with the transition metal, as *B*-site modified, decreased in lead-free ferroelectric Bi-based materials; this phenomenon could be due to the presence of new local states in the electronic structure of both the highest occupied molecular orbital and the lowest unoccupied molecular orbital in the total band structure^9–11,14,57^. In addition, the reduced optical band gap in *A*-site modified Na_0.5_Bi_0.5_TiO_3_-based material was possibly a result of changes in the bonding type between hybridizations *A*-O^58^. Oxygen vacancies created because of unbalanced charges between impurities and hosts (e.g. Fe^2+/3+^ substitute for Ti^4+^, and Ba^2+^ replacement for Bi^3+^) also led to the reduction in the optical band gap because the oxygen vacancy states normally located below and near the conduction band^9–11,59,60^. Thus, we suggest that the random substitution of Ba and Fe ions into the host Na_0.5_Bi_0.5_TiO_3_ could alter the electronic band structure, resulting in reduction of the optical band gap.

**Figure 6:**
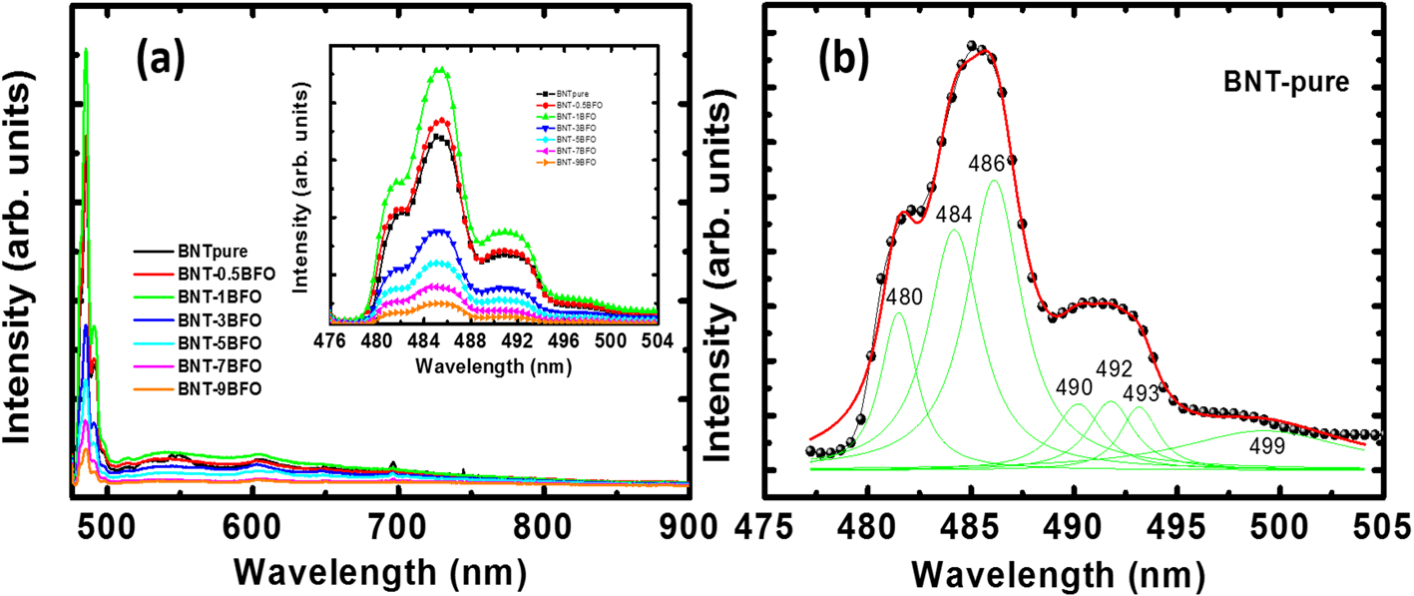
**(a)** PL spectral of pure Bi_0.5_Na_0.5_TiO_3_ materials and BaFeO_3−*δ*_-modified Na_0.5_Bi_0.5_TiO_3_ samples as a function of BaFeO_3−*δ*_ concentration, and **(b)** deconvolution of PL spectral of pure Bi_0.5_Na_0.5_TiO_3_ materials. The inset of **(a)** shown the magnification of PL spectral of pure and BaFeO_3−*δ*_-modified Na_0.5_Bi_0.5_TiO_3_ samples as a function of BaFeO_3−*δ*_ concentration.

Figure 6a shows the room-temperature PL emission spectra of pure Na_0.5_Bi_0.5_TiO_3_ and BaFeO_3−δ_-modified Na_0.5_Bi_0.5_TiO_3_ samples with various BaFeO_3−δ_ amount. The PL spectral of all samples exhibited a broad band emission while strong emission showed in range from 479 to 505 nm. The addition of BaFeO_3−δ_ into host Na_0.5_Bi_0.5_TiO_3_ materials as solid solution suppressed the emission peak, as shown in inset of Fig. 6a. However, we noted that a slight addition of BaFeO_3−δ_ concentration enhanced the emission intensity. In addition, the PL spectral of pure Na_0.5_Bi_0.5_TiO_3_ and BaFeO_3−δ_-modified Na_0.5_Bi_0.5_TiO_3_ samples was overlapped together, suggesting to multi-emission peaks with closed together. Thus, we tried to distinguish the multi-emission peaks via Lorentz fitting. The deconvoluted emission peaks of pure Na_0.5_Bi_0.5_TiO_3_ samples were shown in Fig. 6b. The broad band visible luminescence was also recently reported for ferroelectric titanates-based materials at room temperature such as BaTiO_3_, SrTiO_3_, PbTiO_3_ etc.^61^. The observations in broad band emission were also archived in Bi_0.5_K_0.5_TiO_3_ materials, which were related to the surface effect and/or self-defect effect^56^. Normally, the coordination status of the atoms at the surface of materials is unsaturated, resulting in unpaired states, that make them different from that in the bulk^62^. The unsaturated atoms that existed at the surface region of Na_0.5_Bi_0.5_TiO_3_ materials formed local levels in the forbidden gaps, this displayed the effect of the self-trapped excitons^63^. Therefore, the incident photon was absorbed by the Na_0.5_Bi_0.5_TiO_3_ powder as it is illuminated with the excited source. The absorption photons could create some localized levels and form small polarons. The interaction between the holes in the valence band and polarons formed by the intermediate self-trapped excitons caused blue shift of the luminescence^63^. In addition, the structural distortion because of coupling of TiO_6_-TiO_6_ adjacent octahedra generate the localized electronic levels above the valence band. The recombination from these levels may result in the photoluminescence of Bi_0.5_K_0.5_TiO_3_ materials^62^. The photoluminescence of ferroelectric materials is not generally governed by band-to-band transition, owning to the difficulty in recombination of electron–hole pairs and the separation of the natural polarization domain in the materials. In this kind of materials, the surface states were in charge of the luminescence, in which many unsaturated atoms that presented on the surface of the ferroelectric materials creates the localized levels in the forbidden gaps. Interestingly, the intensity of PL emission of Na_0.5_Bi_0.5_TiO_3_ materials was suppressed by the addition of BaFeO_3−δ_, as shown in the inset of Fig. 6b. The PL emission spectra of BaFeO_3−δ_-modified Na_0.5_Bi_0.5_TiO_3_ materials did not change, indicating the lack of Ba and Fe substitution at the *A*- and *B*-sites, respectively, in the new electron–hole transitions. Thus, the substitution of Fe cation with Ti at the octahedral sites created oxygen vacancies; such vacancies acted as the chapping electron generated from absorbance photon energy, thereby prevented the recombination of the electron–hole pairs to generate photons.

**Figure 7:**
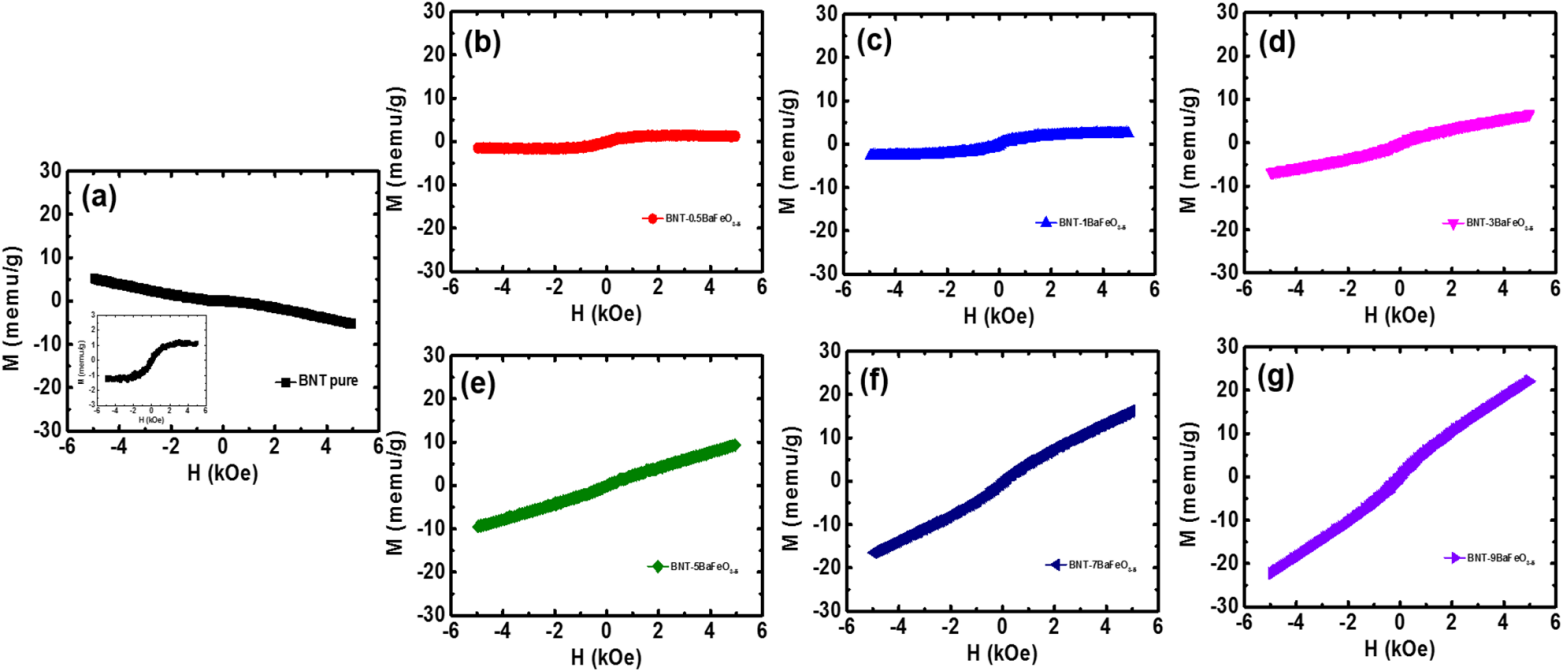
*M*–*H* curves at room temperature of **(a)** undoped Na_0.5_Bi_0.5_TiO_3_, and BaFeO_3−*δ*_-modified Na_0.5_Bi_0.5_TiO_3_ samples with **(b)** 0.5 mol%, **(c)** 1 mol%, **(d)** 3 mol%, **(e)** 5 mol%, **(f)** 7 mol% and **(g)** 9 mol% BaFeO_3−*δ*_ as solid solution. Inset of **(a)** shown the M-H curve of pure Bi_0.5_Na_0.5_TiO_3_ material after substrate diamagnetic components.

Furthermore, the role of BaFeO_3−δ_ solid solution in Na_0.5_Bi_0.5_TiO_3_ materials in imparting magnetism was dependent on the applied magnetic field at room temperature, as shown in Fig. 7a–g for pure Na_0.5_Bi_0.5_TiO_3_ and BaFeO_3−δ_-modified Na_0.5_Bi_0.5_TiO_3_ materials with BaFeO_3−δ_ concentration of 0.5, 1, 3, 5, 7 and 9 mol%, respectively. The pure Na_0.5_Bi_0.5_TiO_3_ exhibited the anti-*S*-shape in *M-H* curves, indicating the combination of diamagnetism and weak ferromagnetism, as shown in Fig. 7a. The diamagnetism in pure Na_0.5_Bi_0.5_TiO_3_ samples originated from the electronic configuration of Ti^4+^ as 3*d*°, whereas the weak ferromagnetism originated from self-defects^9–11,14,16,30,64,65^. The typical hysteresis loop of ferromagnetism was obtained for pure Na_0.5_Bi_0.5_TiO_3_ materials after subtract the diamagnetic components, as shown in inset of Fig. 7a. The saturation magnetization was estimated around 1.5 memu/g which were well consisted with recently reported by Ju et al.^16^. In addition, the remanent magnetization (*M*_r_) and coercive field (*H*_C_) of pure Na_0.5_Bi_0.5_TiO_3_ materials were approximately 0.11 memu/g and 73 Oe, respectively, which were solid evidence for presentation of ferromagnetic state at room temperature. The estimation for their values were also performed with recently reported by Thanh et al. and Ju et al. which those were possibly originated from self-defect such as Na-, Ti- or O-vacancies^10,16^. The *M-H* curves trend to switch from anti-*S*-shape to *S*-shape in the BaFeO_3−δ_-modified Na_0.5_Bi_0.5_TiO_3_ samples as the BaFeO_3−δ_ concentration in the solid solution increase, providing evidence regarding the strength enhancement of ferromagnetic ordering in the samples. As shown in Fig. 7c, the typical ferromagnetic hysteresis loops were obtained where the magnetization trended to saturate as the external applied magnetic field increase, and the strength of ferromagnetic increase. However, further increasing amounts of BaFeO_3−δ_ into host Na_0.5_Bi_0.5_TiO_3_, the *M-H* curves exhibited the unsaturation with low applied external magnetic field, as shown in Fig. 7d–g. The dependence of shape in magnetic hysteresis loop of BaFeO_3−δ_-modified Na_0.5_Bi_0.5_TiO_3_ materials represented that the magnetic properties of BaFeO_3−δ_-modified Na_0.5_Bi_0.5_TiO_3_ materials were very complex, on the one hand the magnetic properties of Na_0.5_Bi_0.5_TiO_3_ materials were strong dependent of the concentration of BaFeO_3−δ_ as solid solution. The *M*_r_ and *H*_C_ values of BaFeO_3−δ_-modified Bi_0.5_Na_0.5_TiO_3_ materials were approximately 51–106 Oe and 0.12–0.48 memu/g, respectively. These results were consistent with the recently observed *M*_r_ and *H*_C_ of transition-metal-doped lead-free and lead-based ferroelectric materials^8–11,60,64–68^. The nonzero *M*_r_ and *H*_C_ values of BaFeO_3−δ_-modified Bi_0.5_Na_0.5_TiO_3_ materials provided solid evidence for the presence of the ferromagnetic state at room temperature. In additon, the maximum magnetization was estimated around 23 memu/g for 9 mol% BaFeO_3−δ_ solid solution in host Na_0.5_Bi_0.5_TiO_3_ materials. That value was larger than that of self-defect induced magnetism of pure Na_0.5_Bi_0.5_TiO_3_ materials or single transition metals doped Na_0.5_Bi_0.5_TiO_3_ materials, in which around ~ 1.5 memu/g for Cr-doped Na_0.5_Bi_0.5_TiO_3_, ~ 3 memu/g for Co-doped Na_0.5_Bi_0.5_TiO_3_, ~ 9 memu/g for Mn-doped Na_0.5_Bi_0.5_TiO_3_, ~ 15 memu/g for Fe-doped Na_0.5_Bi_0.5_TiO_3_ materials, and ~ 4 memu/g for Ni-doped Na_0.5_Bi_0.5_TiO_3_ materials^8–11,60,68^. Herein, we need to remark that the origin of ferromagnetism ordering of transition metal impurities containing Na_0.5_Bi_0.5_TiO_3_ materials at room temperature were still debated. The weak-ferromagnetism in pure Na_0.5_Bi_0.5_TiO_3_ materials were possibly originated from self-defect and/or surface defect (such as Ti and Na-vacancies) while the magnetization of Na_0.5_Bi_0.5_TiO_3_ materials were slightly enhanced via oxygen vacancies^11,14,16,30^. The Mn-, Ni- and Fe-doped Na_0.5_Bi_0.5_TiO_3_ materials exhibited the room temperature ferromagnetism which were related to intrinsic phenomenon where the transition cations such of Mn, Ni and Fe interacted with the oxygen vacancies, like *F-*center interaction mechanism, e.g. Mn^2+/3+^–¤–Mn^2+/3+^ or Fe^2+/3+^–¤–Fe^2+/3+^ pairs etc., which were favored for ferromagnetic ordering^8–11,60,64^. Unlikely Mn-, Ni- and Fe- cations impurities in Na_0.5_Bi_0.5_TiO_3_ materials, the Co impurities trended to form Co-clusters embedding in host Na_0.5_Bi_0.5_TiO_3_ materials which displayed the room temperature ferromagnetism^8^. Recently, our experimental observation along with first principle calculation predicted that the interaction of Co cations into host Na_0.5_Bi_0.5_TiO_3_ materials possibly displayed the weak ferromagnetism at room temperature^64^. In addition, Hung et al. reported that MgFeO_3−δ_ solid solution in Na_0.5_Bi_0.5_TiO_3_ materials exhibited strong magnetization, which were estimated to be around 39.6 memu/g, where the Mg cations played an importance role for mediating ferromagnetism^28^. The SrFeO_3−δ_- and CaFeO_3−δ_-modified Na_0.5_Bi_0.5_TiO_3_ materials also showed strong enhancement of the magnetization at room temperature^29,30^. Note that the Mg cations possibly substituted for both *A*-site (Bi^3+^, Na^+^) and *B*-site in Na_0.5_Bi_0.5_TiO_3_ crystal structure while Sr and Ca cations only replaced with *A*-site in host Na_0.5_Bi_0.5_TiO_3_ crystal structure^28–30^. Thus, we suggested that the possible room temperature ferromagnetism in BaFeO_3−*δ*_-modified Na_0.5_Bi_0.5_TiO_3_ materials were strongly related to the interaction of Fe cations through oxygen vacancies, like *F*-central interaction, which were recently suggested for Mn-, Ni-, Co- and Fe-doped Na_0.5_Bi_0.5_TiO_3_ materials^9,10,60,64,69^. A recent X-ray photoelectron spectroscopy (XPS) analysis of CaFeO_3−δ_-modified Na_0.5_Bi_0.5_TiO_3_ materials showed that Fe cations are stable in the Fe^2+^ and Fe^3+^ valence state together with O vacancies^30^. Therefore, we suggest that the interaction pair Fe^2+/3+^–¤–Fe^2+/3+^ favors ferromagnetic ordering^69^. The unsaturation in the *M–H* curves of magnetic hysteresis loops of Na_0.5_Bi_0.5_TiO_3_ materials (under 6 kOe of applied magnetic field) as the amount of the BaFeO_3−δ_ solid solution increase suggested magnetic polaron interaction, wherein the interaction between Fe^2+/3+^–¤–Fe^2+/3+^ versus Fe^2+/3+^–¤–Fe^2+/3+^ resulted in antiferromagnetic-like ordering^9,69–73^. In addition, isolated Fe cations displayed paramagnetic properties^9,69–73^. Thus, the combination of the complex signal of ferromagnetic interaction and antiferromagnetic-like and paramagnetic properties was observed when the BaFeO_3−δ_ solid solution was present at high concentrations in host Na_0.5_Bi_0.5_TiO_3_ materials. However, unlike single Fe-doped Na_0.5_Bi_0.5_TiO_3_ materials, the modification at *A*-site (Bi^3+^, Na^+^) via Ba^2+^ cations in host Na_0.5_Bi_0.5_TiO_3_ materials also possibly contributed a source to the ferromagnetism ordering, in which the substitution of Ba^2+^ cations for Bi^3+^ cations in crystal structure created the O-vacancies while Ba^2+^ cations incorporated for Na^+^ cations generated the Na-vacancies. Both O- and Na- vacancies are origin of the ferromagnetism, but they work in different ways^16,30^. Nevertheless, both the experimental observation and theoretical prediction have agreed that Na-vacancies induce the nonzero magnetic moment^16,30^. Therefore, the strength of magnetic moments can be increased by increasing the number of Na-vacancies. However, unlikely Na-vacancies, the O-vacancies were predicted to be agent of the nonmagnetic moment^30^. The O-vacancies were important in promoting the reduction in valence state from Ti^4+^ to Ti^3+^ (even Ti^2+^) because of oxygen vacancies bounding surround^30,31,74–76^. The theorey predicted that Ti^4+^ has no magnetic moment whereas the Ti^3+^ or Ti^2+^ have nonzero magnetic moment^30^. Thus, the enhancement in O-vacancies were indirectly induced by the magnetic moment along with the contribution of magnetization of Ti^3+/2+^ defects, resulting in increased magnetic moment that were over the self-defects compared with pure Na_0.5_Bi_0.5_TiO_3_ samples. Recently, XPS results have shown that the Ti^4+^ cations in Na_0.5_Bi_0.5_TiO_3_ materials were possible reduced to Ti^3+^ via modification of CaFeO_3−δ_- and SrMnO_3−δ_-modified as solid solution^30,31^. Therefore, we suggested that the increasing the O-vacancies was promoted by the magnetization of self-defect of Ti^3+^ cations. Recently, the interaction between Co^3+/2+^ cations and reduction of valence state of Ti^4+-δ^ pair through O-vacancies were suggested to favor the ferromagnetic ordering^35^. Therefore, we suggested that the appearance of interaction Fe^2+/3+^–¤–Ti^4+-δ^ pair may arouse increasing of the strength of ferromagnetic ordering. We recently reported that the magnetic properties of various magnetic compound, such as Mn_2_O_3_, Mn_5_Ge_3_ etc., could be tunable by a strain^77,78^. Therefore, due to the difference radius with ions of host lattice, the Ba cations possibly caused a chemical pressure, possibly resulting in the change of interaction between them through oxygen vacancies; finally, resulted in modification of magnetic ordering strength. Recent experimental and theoretical studies have suggested that transition metals may fill *A*- and *B*-sites in the perovskite structure of lead-free ferroelectrics, thus resulting in complex magnetic properties^27,79–83^. Liu et al. reported that K_0.45_Na_0.49_Li_0.06_NbO_3_ materials modified with Cu at the *A-*site show paramagnetic properties^80^. However, Yang et al. reported that Cu modification at the *B*-site of EuMnO_3_ materials changes magnetic properties from paramagnetic to antiferromagnetic ordering^81^. Deng et al. found that the paramagnetic properties of EuMnO_3−δ_ materials were changed to antiferromagnetic via modification at the Eu site by Mn cations^82^. Magnetic phase transition from paramagnetic to antiferromagnetic properties has also been reported for DyMnO_3_ materials modified with the Mn cation at the Dy-site^83^. Theoretical and experimental studies have suggested that CoTiO_3_-modified Na_0.5_Bi_0.5_TiO_3_ materials have complex magnetic properties that are strongly dependent on the location of Co impurities at the *A*-site or *B*-site in the host structure^27,79^. Notably, the radii of Fe^2+/3+^ cations with VIII coordination are 0.92 Å and 0.78 Å, respectively, which are comparable with the radii of Bi^3+^ (1.17 Å) and Na^+^ (1.39 Å) cations^84^. Therefore, we suggested that Fe cations were random incorporated at both the *A*-site and *B*-site likely contributing to the complex magnetic properties of the host Bi_0.5_Na_0.5_TiO_3_ materials. The role of Fe cation substitution at the *A*- and *B*-sites in the magnetic properties of Na_0.5_Bi_0.5_TiO_3_ materials was further investigated by using density-functional theory (DFT) calculation.

**Figure 8:**
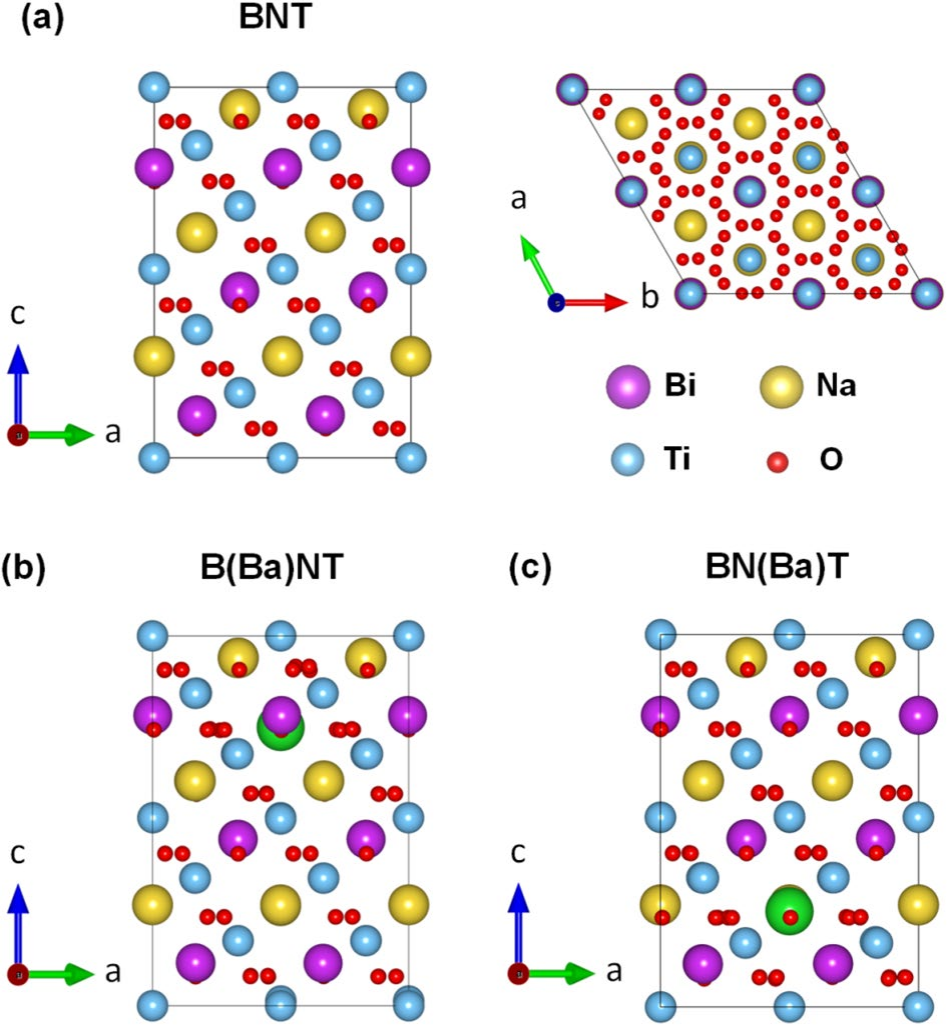
**(a)** Side and top views of the optimized atomic structure of Na_0.5_Bi_0.5_TiO_3 _(BNT). The same with **(b)** Bi-site Ba [B(Ba)NT] and **(c)** Na-site Ba [BN(Ba)T] substitution.

**Figure 9:**
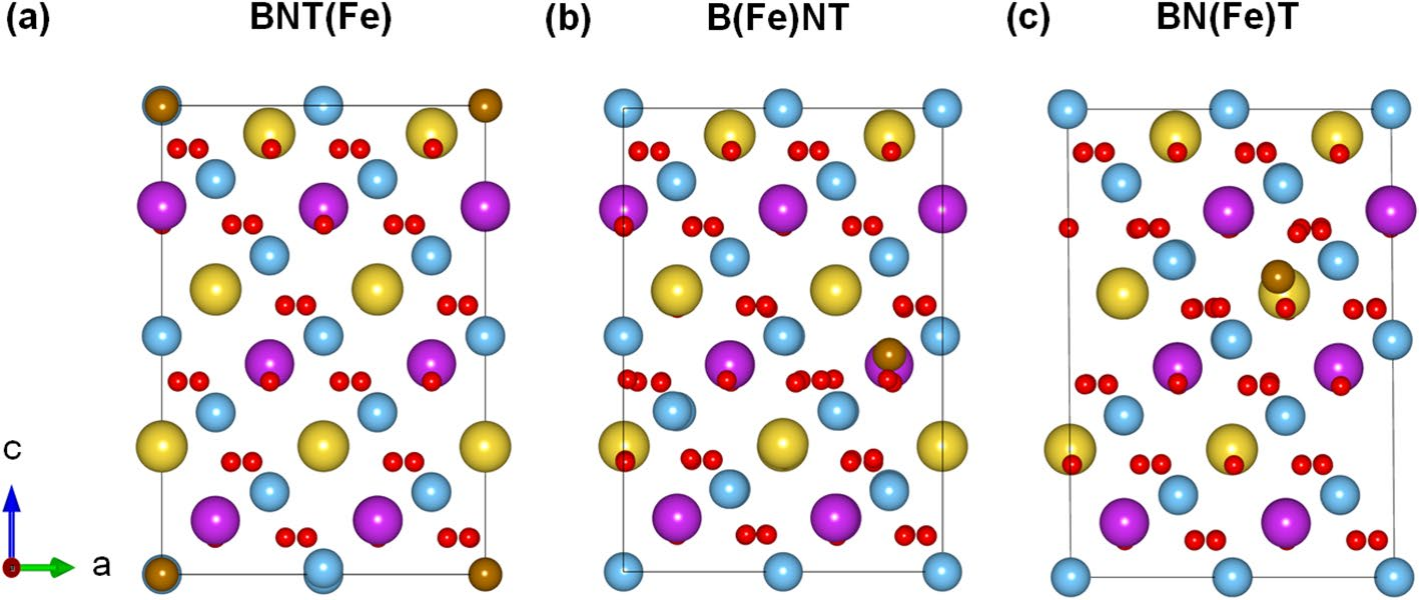
**(a)** Side views of the optimized atomic structure of the **(a)** Ti-site [BNT(Fe)], **(b)** Bi-site [B(Fe)NT], and **(c)** Na-site Fe [BN(Fe)T] substitution. The atomic symbols follow the same convention used in Fig. 8. Brown spheres are the Fe substitutional atoms.

To elucidate the origin of the observed ferromagnetism in BaFeO_3−*δ*_-doped Na_0.5_Bi_0.5_TiO_3_, the density-functional theory (DFT) calculations were performed using the Vienna ab initio Simulation Package (VASP)^85,86^. The generalized gradient approximation (GGA) formulated by Perdew, Burke, and Ernzerhof (PBE) was used for the electron exchange correlation potential^87^. Figure 8a shows the side and top views of the rhombohedral crystal structure of the 24 formula unit (f.u.) cell (120-atom) adopted for Bi_0.5_Na_0.5_TiO_3_ (BNT). As model systems shown in Fig. 8b,c, we have considered the substitution of one Ba atom for the Bi-site, denoted as B(Ba)NT, and Na-site [BN(Ba)T], in the 24 f.u. cell structure of the BNT. This corresponds to about 0.83 at.% doping for the A-site (Bi and Na) substitution, which is within the range of the present experimental doping concentrations (0.5–9 mol.%). To represent the presence of the Fe substitution in a sample, we replaced one Ti [BNT(Fe)], Ba [B(Fe)NT], and Na [BN(Fe)T] atom with the Fe atom in the same unit cell, as shown in Fig. 9a–c. For all systems, we used an energy cutoff of 500 eV for the plane-wave basis and a *k*-point mesh of 5 × 5 × 5 for the Brillouin zone integration. To obtain optimized atomic structures, the atomic positions as well as lattice parameters were fully relaxed until the largest force becomes less than 10^−2^ eV*/*Å and the change in the total energy between two ionic relaxation steps is smaller than 10^−5^ eV. Note that the severe distortions of octahedral TiO_3_ lattice were observed for all geometries after optimization.

We first investigate the energetics of the Ba- and Fe-doped BNT. Here, the formation energy (*H*_*f*_) is defined as ${H}_{f}=H-\sum_{i}{\mu }_{i}{n}_{i}$, where *H* is the total energy of the system, and ${\mu }_{i}$ and ${n}_{i}$ are the chemical potential and the number of species *i* in the unit cell. The calculated *H*_*f*_ values of BNT, B(Ba)NT, BN(Ba)T, B(Fe)NT, BN(Fe)T, and BNT(Fe) are shown in Table 1. We find that the *H*_*f*_ of BNT is -2.385 eV/atom, which indicates the pure BNT is quite stable. Our calculations further indicate that the Ba substitute prefers either the Bi or Na sites (*A*-site), as their enthalpies of formation are competitive (− 2.402 and − 2.392 eV/atom). The Fe dopant atoms may also occupy both the *A*- and *B*-site (Ti), although the absolute values of *H*_*f*_ for the *A*-site (− 2.376 eV/atom for the Bi and − 2.355 eV/atom for the Na) are higher than that (− 2.335 eV/atom) of the *B* (Ti)-site in magnitude. Nevertheless, in a real sample, the latter substitution (*B*-site) might appear more than the *A*-site (Bi and Na) substitution, as the *A*-site is mainly occupied by the Ba atoms.

**Table 1:** The DFT results of the formation energy *H*_*f*_ (eV/atom), magnetic energy ΔE (eV/atom), and magnetization *M* (μ_B_/f.u.) of BNT, B(Ba)NT, BN(Ba)T, B(Fe)NT, BN(Fe)T, and BNT(Fe) compounds. We also list the magnetic moment *m* (μ_B_/atom) of the Fe dopant atom for the 1 Fe atom doped 24 f.u. cell of B(Fe)NT, BN(Fe), and BNT(Fe).

	*H*_*f*_ (eV/atom)	Δ*E* (eV)	*M* (μ_B_/f.u.)	*m* (μ_B_/atom)
BNT	−2.385	0	0	–
B(Ba)NT	−2.402	0	0	0
BN(Ba)T	−2.392	0	0	0
B(Fe)NT	−2.376	1.15	0.21	3.94
BN(Fe)T	−2.355	1.20	0.14	3.42
BNT(Fe)	−2.335	0.73	0.16	3.10

Table 1 shows the calculated magnetic energy (Δ*E* = *E*_sp_ – *E*_non-sp_, where *E*_sp_ and *E*_non-sp_ are the total energies of the spin-polarized and non-spin-polarized states, respectively), total magnetization per f.u. (*M*), and atom resolved magnetic moment (*m*) of the dopant atoms. Our calculations show that all the Fe-doped structures are magnetic, while the Ba-doped ones are nonmagnetic. The calculated magnetization ranges from 0.14 to 0.21 µ_B_/f.u. (or approximately 3.6 to 5.5 emu/g with [emu/g] = 1.078⋅10^20^ (*M*_f.u._/*N*_A_) [µ_B_/f.u.], where the *N*_A_ and *M*_f.u._ are the Avogadro constant and the molar mass per f.u., respectively) at 0.83 at. % doping. These theoretical magnetization values are much higher than the maximum experimental value of 23 memu/g for 9 mol.% BaFeO_3−δ_-modified Na_0.5_Bi_0.5_TiO_3_. The induced magnetization mainly comes from the local atomic moment of the Fe dopant atoms, as shown in Table 1.

**Figure 10:**
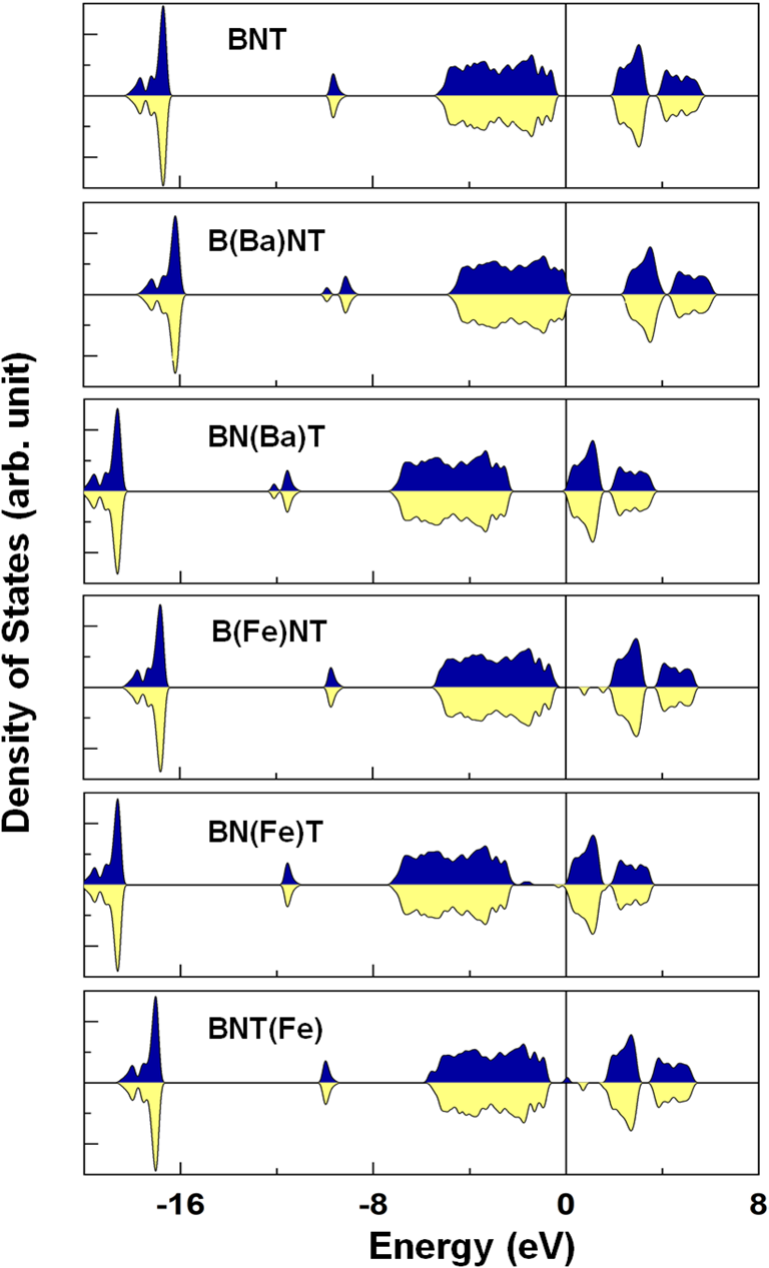
Top to bottom: The DFT results of the spin-resolved DOS for the BNT, B(Ba)NT, BN(Ba)T, BNT(Fe), B(Fe)NT, and BN(Fe)T. The Fermi level is set to zero energy.

**Figure 11:**
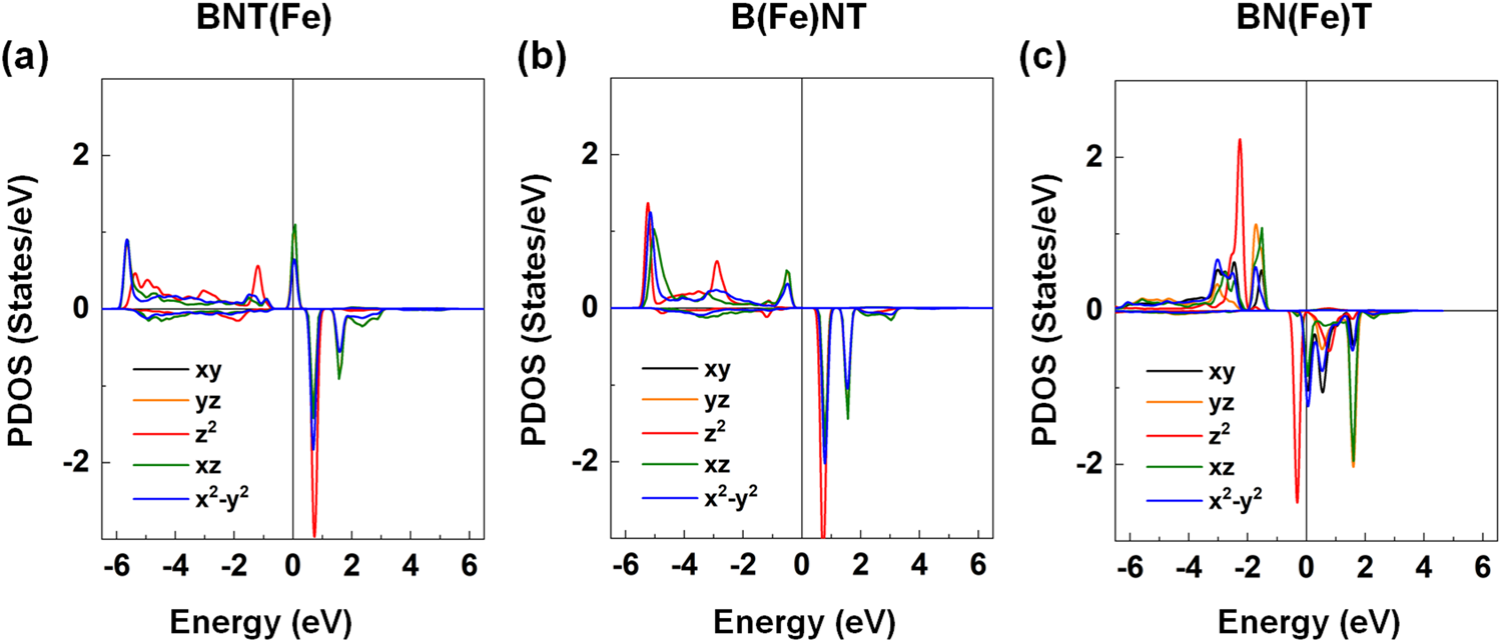
The DFT results of the *d*-orbital decomposed PDOS of the Fe substitutional atom for the **(a)** BNT(Fe), **(b)** B(Fe)NT, and **(c)** BN(Fe)T. The black, orange, red, green, and blue lines represent the *d*_xy_, *d*_yz_, *d*_z_^2^, *d*_xz_, and *d*_x_^2^_–y_^2^ orbital states, respectively. The Fermi level is set to zero energy.

Figure 10 presents the spin-resolved density of states (DOS) of BNT, B(Ba)NT, BN(Ba)T, B(Fe)NT, BN(Fe)T, and BNT(Fe) compounds. We have also analyzed the orbital projected DOS (PDOS) of the Fe 3*d* orbital states in Fig. 11 for the selected B(Fe)NT, BN(Fe)T, and BNT(Fe) as they exhibit magnetic nature. For BNT, the valence and conduction bands are characterized by the O-2*p* and Ti-3*d* orbital states with a band gap of ~ 2.25 eV, respectively. The majority- and minority-spin states are entirely degenerate, which indicates a feature of nonmagnetic ground state. The calculated band gap of BNT is smaller than the measured value (3.08 eV), which is quite typical in DFT calculations for oxide perovskites^88^. The Bi-site Ba gives rise to the upward shift of the valence band states toward the Fermi level. The opposite appears for BN(Ba)T, where the Fermi level shifts upward and touches the minimum of the conduction bands. Thus, the former and latter systems are referred to as *p*- and *n*-doped semiconductors, while kept the absolute value of the band gap. On the other hand, as shown in the bottom panels in Fig. 10, for B(Fe)NT, BN(Fe)T, and BNT(Fe), there are some midgap states around the Fermi level. In particular, for BNT(Fe), a finite DOS peak state appears right at the Fermi level in the majority-spin state while the other spin channel exhibits an insulating behavior. This is a feature of the half-metallic electronic nature. Furthermore, for all the Fe-doped compounds, substantially large exchange splitting between the spin subbands (i.e., majority-spin and minority-spin) is prominent (Fig. 10). These peak states are due to the strong orbital hybridization between the Fe 3*d* and O 2*p* states. As shown in Fig. 11, the majority-spin bands of the Fe are fully occupied, and the minority-spin states are almost unoccupied for the BNT(Fe) and B(Fe)NT but partially occupied for the BN(Fe)T. Overall, one can expect the large magnetic moment at the Fe site, as addressed in Table 1. Induced moments at the neighboring sites to the Fe are rather small.

Based on the PDOS analyses for the BNT(Fe) and B(Fe)NT compounds, we infer that the six (five) *d*-orbitals of Fe^2+^ (Fe^3+^) ion split by high-spin state through the crystal field theory are filled by the 5 majority-spin electrons in the low-lying *t*_2g_ orbital levels and 1 electrons (no electron) in the minority-spin *t*_2g_ state. Thus, according to Hund's rule, the calculated magnetic moments of 4 and 5 μ_B_ of the Fe replacement for the Ti and Bi sites can be explained by the electronic configuration of the high-spin state in the crystal field theory through the unpaired electron spin count. For the BN(Fe)T, the magnetic moment of the Fe atom is reduced compared with those for the other two systems, as some minority-spin states are partially occupied (Fig. 11c). Furthermore, for all compounds, both the *t*_2g_ and *e*_g_ states in PDOS are slightly split, which is mainly due to the Jahn–Teller effect as the severe octahedron distortion occurs in the presence of the Fe substitution.

**Figure 12:**
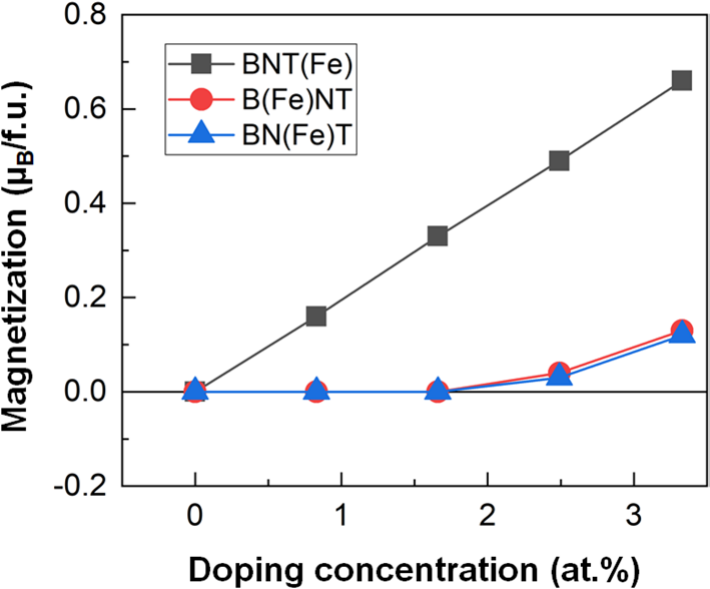
The DFT results of the magnetization (µ_B_/f.u.) of BNT(Fe), B(Ba)NT, and BN(Ba)T as function of the concentration of the dopant atoms.

We now investigate the doping concentration dependent magnetization of the Fe and Ba doped BNT. Figure 12 shows the calculated *M* of BNT(Fe), B(Fe)NT, and BN(Fe)T as function of the concentration of the dopant atoms. For the 2 (or 1.67 at.%) and 4 (or 3.33 at.%) Fe atoms in the 24 f.u. cell, we have also considered the spin antiparallel coupling between the Fe dopant atoms. For both cases, the spin parallel coupling (ferromagnetic) is more preferable than the spin antiparallel coupling.

**Figure 13:**
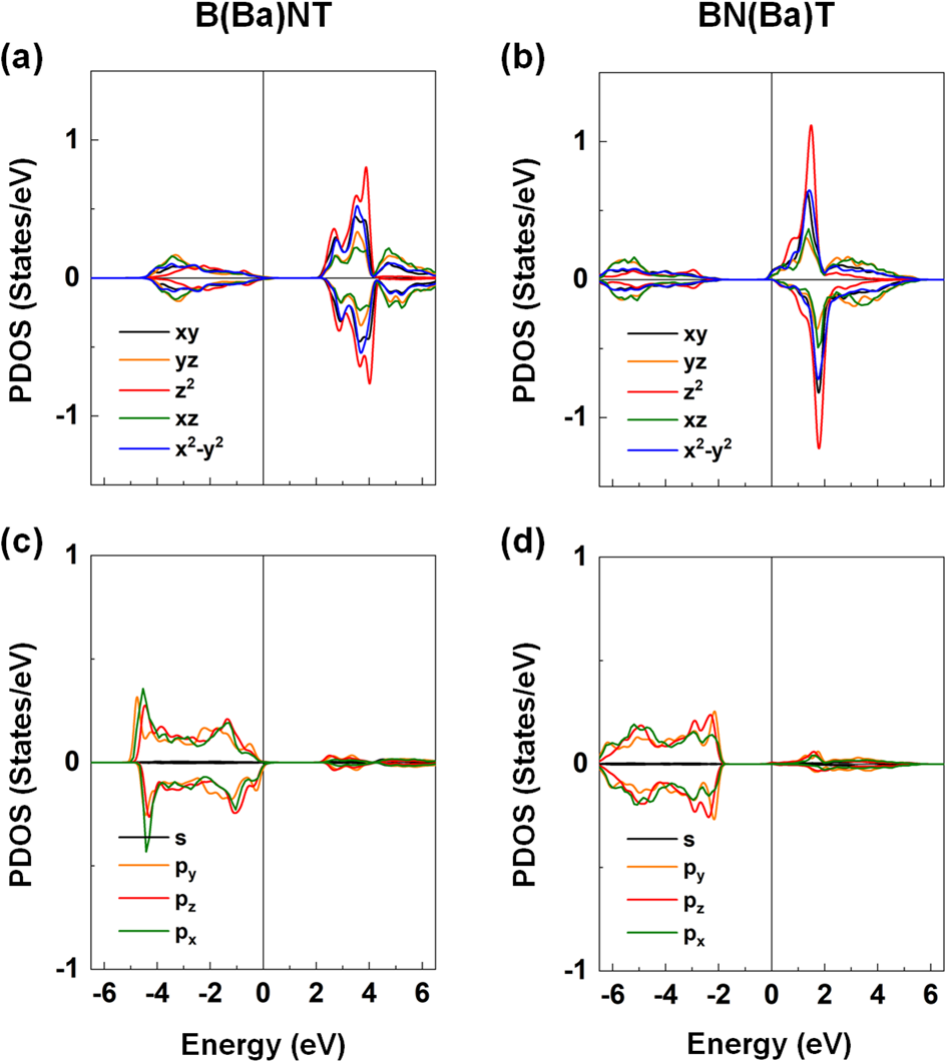
The DFT results of PDOS of the Ti and O atoms for **(a)** and **(c) **(Ba)NT and **(b)** and **(d)** BN(Ba)T at 3.33 at./% Ba doping. The Fermi level is set to zero energy.

Clearly, the magnetization increases linearly from 0.16 µ_B_/f.u. (4.2 emu/g) to 0.66 µ_B_/f.u. (17.3 emu/g) as the Fe concentration increases from 0.83 at.% to 3.33 at.%, as shown in Fig. 12. Our calculated magnetization is much larger than the measured values (23 memu/g at 9 mol.%), which is presumably due to the different concentrations and stoichiometries between the theory (here only the Fe doping) and experiment (Ba and Fe co-doping). Interestingly, the Ba doping for the *A*-site (Bi and Na) induces magnetism (*M* = 0.05 µ_B_/f.u.) at about 2.5 at.% (Fig. 12). It is further found that the calculated magnetization increases as the doping concentration increases. Our atom resolved magnetization analyses indicate that the induced magnetization mainly comes from the Ti and O atoms neighboring to the Ba dopant site. The underlying mechanism can be explained by the spin-polarized charge transfer between the dopant and neighboring atoms in the unit cell, as revealed from the Ti- and O-PDOS analyses shown in Fig. 13.

**Figure 14:**
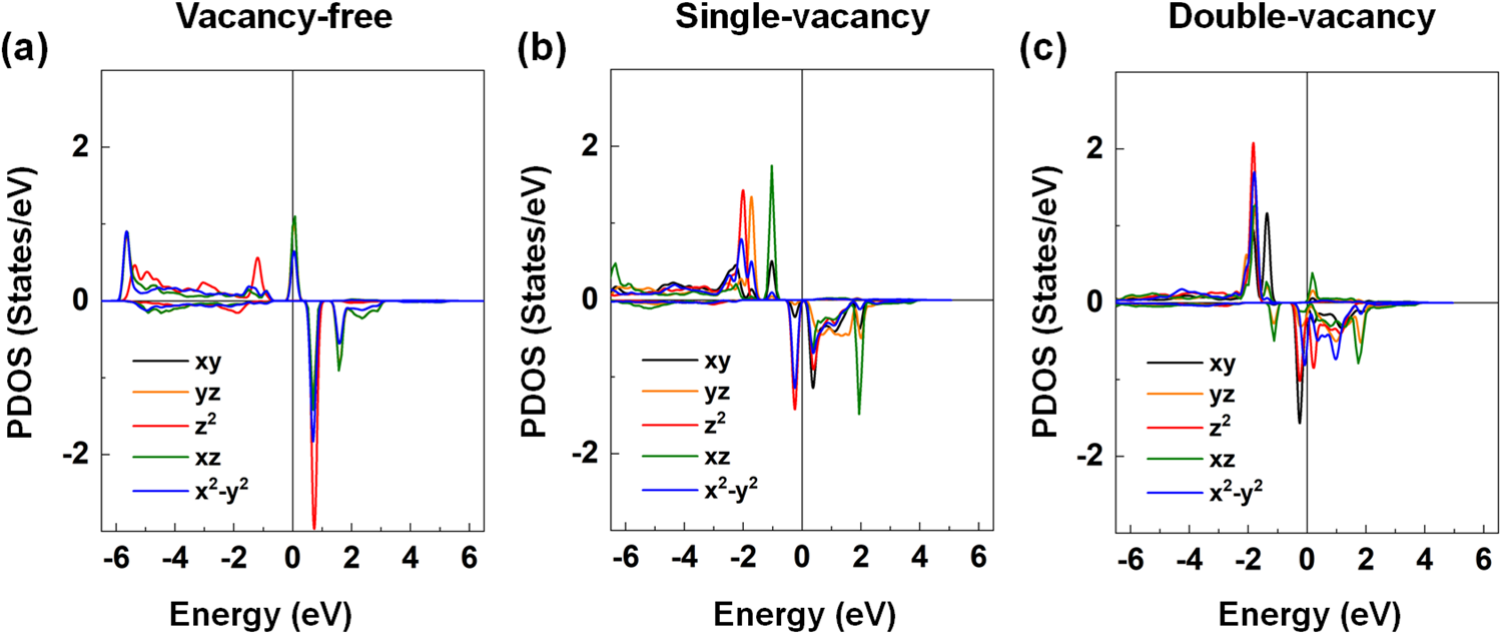
The DFT results of the *d*-orbital decomposed PDOS of the Fe substitutional atom for the BNT(Fe) with the **(a)** vacancy-free, **(b)** single-vacancy, and **(c)** double-vacancy. The black, orange, red, green, and blue lines represent the *d*_xy_, *d*_yz_, *d*_z_^2^, *d*_xz_, and *d*_x_^2^_–y_^2^ orbital states, respectively. The Fermi level is set to zero energy.

We finally explored the magnetic and electronic properties of the O-vacancy defected BNT(Fe). We have considered the presence of a single vacancy and double vacancies in the Fe-included octahedral cell to imitate different valence states of the Fe dopant atom. Our calculations show that the total magnetization (4 μ_B_) of the BNT(Fe) compound decreases by 1 and 2 μ_B_ for the single-vacancy and double-vacancy systems, respectively. From the electronic structure analyses shown in Fig. 14a–c, the PDOS of the Fe dopant atom in BNT(Fe) shift toward the low energy region and some minority-spin states are partially occupied in the presences of the single and double oxygen vacancies. This is because of the extra electrons accumulated at the Fe site, originated from the O deficiency in the unit cell. Furthermore, the obtained magnetic moments are simply the reflections of the Fe^2+^ and Fe^3+^ ionic states in the high spin states and mixture of them in a real sample, as addressed in the previous  experiment^30^.

## Conclusion

The solid solution of BaFeO_3−δ_ and Na_0.5_Bi_0.5_TiO_3_ ceramics have been successfully synthesized by a chemical route sol–gel method. The Ba and Fe ions were distributed randomly into the Na_0.5_Bi_0.5_TiO_3_ lattices which caused in the distortion of lattice structure and decreased the optical band gap. The complex magnetic properties were observed in this solid solution. This work shows a simple way for enhancement of room-temperature ferromagnetism in lead-free ferroelectric materials by solid solution.

## Experiment

(1 − *x*)Na_0.5_Bi_0.5_TiO_3_ + *x*BaFeO_3−δ_ (BNT-*x*BFO; *x* = 0.5%, 1%, 3%, 5%, 7% and 9%) samples were fabricated by sol–gel method. The raw materials were consisted of Bi(NO_3_)_3_^.^5H_2_O, NaNO_3_, Fe(NO_3_)_3_^.^9H_2_O, tetraisopropoxytitanium (IV) (C_12_H_28_O_4_Ti) and BaCO_3_. The solution was chosen which are acetic acid (CH_3_COOH) and deionized water with volume ratio of $V_{H_{2}O}$:$V_{CH_{3}COOH}$ = 5:2 while an acetylacetone (CH_3_COCH_2_COCH_3_) were selected as ligand. Fist, the BaCO_3_ were weighted and distinguee under mix acid acetic and deionized water. Thus, the raw materials such Bi(NO_3_)_3_.5H_2_O, NaNO_3_, Fe(NO_3_)_3_.9H_2_O were weighted to add the solution. To following, the solution was added with tetraisopropoxytitanium after adding to avoid hydrolysis. The solution was magnetic stirred under several hours to make homogeneous solution of sol. The sol was dried under 100 °C to prepare gels in oval. The dried gel was rout grounded and annealed under 800 °C for three hours in air then nature cooling down to room temperature. The as-prepared samples were rout ground for further samples characterization.

The chemical composition and chemical mapping of samples was carried out via energy dispersive spectroscopy (EDS, S-4800 Hitachi). The sodium is lighter element which were easy evaporated during the gelling and annealing processing that make samples nonstoichiometric composition. Therefore, the sodium nitrate was weighed to extra around 40–50 mol% to prevent the sodium evaporation^9–11,24–42,60^. The crystalline structural of pure Na_0.5_Bi_0.5_TiO_3_ and BaFeO_3−δ_-modified Na_0.5_Bi_0.5_TiO_3_ samples were characterized through X-ray diffraction (XRD, Brucker D8 Advance). The vibration modes of samples were measured by using Raman spectroscopy (with a 475 nm LASOS laser and a DU420A-Oe detector). The optical properties were studied by Ultraviolet–Visible (UV–Vis, Jasco V-670) and photoluminescence (PL, excited with 475 nm LASOS laser and a DU420A-Oe CCD detector) spectroscopy. Magnetic properties were characterized by a vibrating sample magnetometer (VSM, Lakeshore 7404). All experimental were performed at room temperature.
